# Descemet’s membrane endothelial keratoplasty in aphakic and vitrectomized eye

**DOI:** 10.3205/oc000129

**Published:** 2020-02-14

**Authors:** Remzi Karadag, Veysel Aykut, Fehim Esen, Halit Oguz, Ahmet Demirok

**Affiliations:** 1 Department of Ophthalmology, Faculty of Medicine, Istanbul Medeniyet University, Goztepe, Istanbul, Turkey

**Keywords:** aphakia, bullous keratopathy, Descemet’s membrane endothelial keratoplasty, DMEK, donor stromal tissue, vitrectomized eye

## Abstract

**Objective:** To report a new technique for Descemet’s membrane endothelial keratoplasty (DMEK) in aphakic and vitrectomized eyes.

**Case description:** A 56-year-old man presented with corneal decompensation in the left eye after combined pars plana vitrectomy and lensectomy for the management of a traumatic eye injury. DMEK graft and recipient bed were prepared as regular. The posterior half of the donor stroma was dissected with a crescent knife to form a temporary stromal barrier tissue. The prepared donor stromal tissue was implanted into the anterior chamber (AC) to form a barrier over the iris and pupilla. Then, the endothelial graft was safely injected into the anterior chamber and deployed by gently tapping on the corneal surface. Air tamponade was applied into the AC for 10 minutes to allow the graft to attach. Afterwards, the stromal barrier tissue was removed through the main incision and the AC was refilled with air tamponade. There were no intraoperative or postoperative complications during 1-month follow-up.

**Conclusion:** Insertion of a temporary posterior stromal tissue as a barrier over the iris and pupilla successfully provided AC stability and prevented posterior dislocation of the graft or air tamponade. This new technique was a safe and effective approach for DMEK in aphakic and vitrectomized eyes.

## Introduction

Descemet’s membrane endothelial keratoplasty (DMEK) is a relatively new posterior lamellar keratoplasty technique that allows the surgeon to replace only the removed dysfunctional endothelial tissue layer with a new one without changing the corneal stroma. As the DMEK graft includes only Descemet’s membrane and the endothelium [1], it is the thinnest graft ever transplanted to the human body. The most challenging part of the DMEK surgery is the unfolding process of the graft in the anterior chamber without touching the endothelial tissue [1], [2]. For this to happen, the surgeon needs to be able to control the depth of the anterior chamber and induce proper fluid currents to open the endothelial graft tissue without touching. Therefore, the presence of an intact iris-lens diaphragm and vitreous humour is essential as a posterior support for the anatomic stability of the anterior chamber. DMEK in aphakic and vitrectomized eyes is especially challenging due to three problems: First and most important, the above-motioned delicate control of the anterior chamber depth is impaired by the absence of a lens and vitreous. Second, there is a potential risk of intraoperative posterior dislocation of the graft, which is unlikely in regular cases. Third, the formation of an effective air or gas tamponade is also challenging in these cases [1], [2], [3]. In order to overcome these problems, we designed a new technique for DMEK in aphakic and vitrectomized eyes. The posterior half of the donor stroma was dissected with a crescent knife and temporarily placed over the iris tissue during the surgery for better anterior chamber stability and to form a barrier. 

## Case description

**Figure 1:**
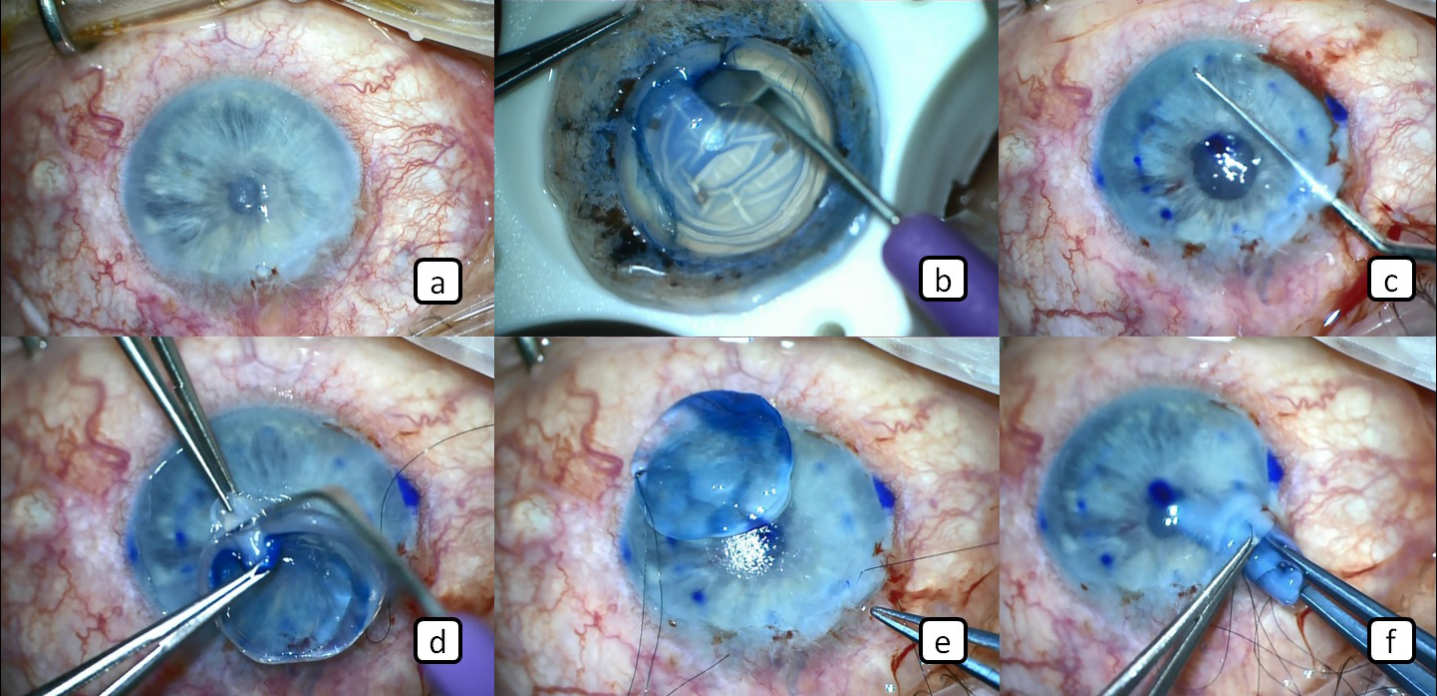
a) Corneal edema in the entire cornea. b) Descemet’s membrane was stripped from the posterior stroma. c) The central 8 mm of the recipient Descemet’s membrane was stripped and removed. d) The posterior stroma of the donor cornea was cut off in half thickness. e) A security suture was placed on the edge of the posterior donor stroma. f) Posterior donor stromal tissue was implanted into the anterior chamber through the main corneal wound to form a secure barrier during the surgery.

A 56-year-old male patient presented with corneal decompensation in the left eye after combined pars plana vitrectomy and lensectomy for the management of a traumatic eye injury. The best corrected visual acuity in the left eye was counting fingers at 1 meter. Slit lamp examination revealed marked corneal edema throughout the entire corneal tissue (Figure 1a (Fig. 1)). As the retinal examination could not be performed with indirect ophthalmoscopy due to media opacity, we performed b-scan ultrasonography and confirmed that the retina was attached. DMEK surgery was scheduled for the management of endothelial failure. 

### Donor tissue preparation

Descemet’s membrane was stripped from the posterior donor cornea and a circular cut was performed with a 8.0 mm trephine to prepare the endothelial graft (Figure 1b (Fig. 1)). The graft was then kept within the Optisol medium until the recipient bed was prepared. The remaining stroma of the donor tissue was cut in half thickness with a crescent knife from the endothelial side (Figure 1d (Fig. 1)). The anterior part of the stroma containing the epithelium was removed and disposed. A safety suture was placed on the edge of the posterior donor stromal tissue with 10-0 nylon in order to ensure that this tissue will not accidentally fall into the vitreus cavity during the surgery (Figure 1e (Fig. 1)).

### The preparation of the recipient bed and the procedure

**Figure 2:**
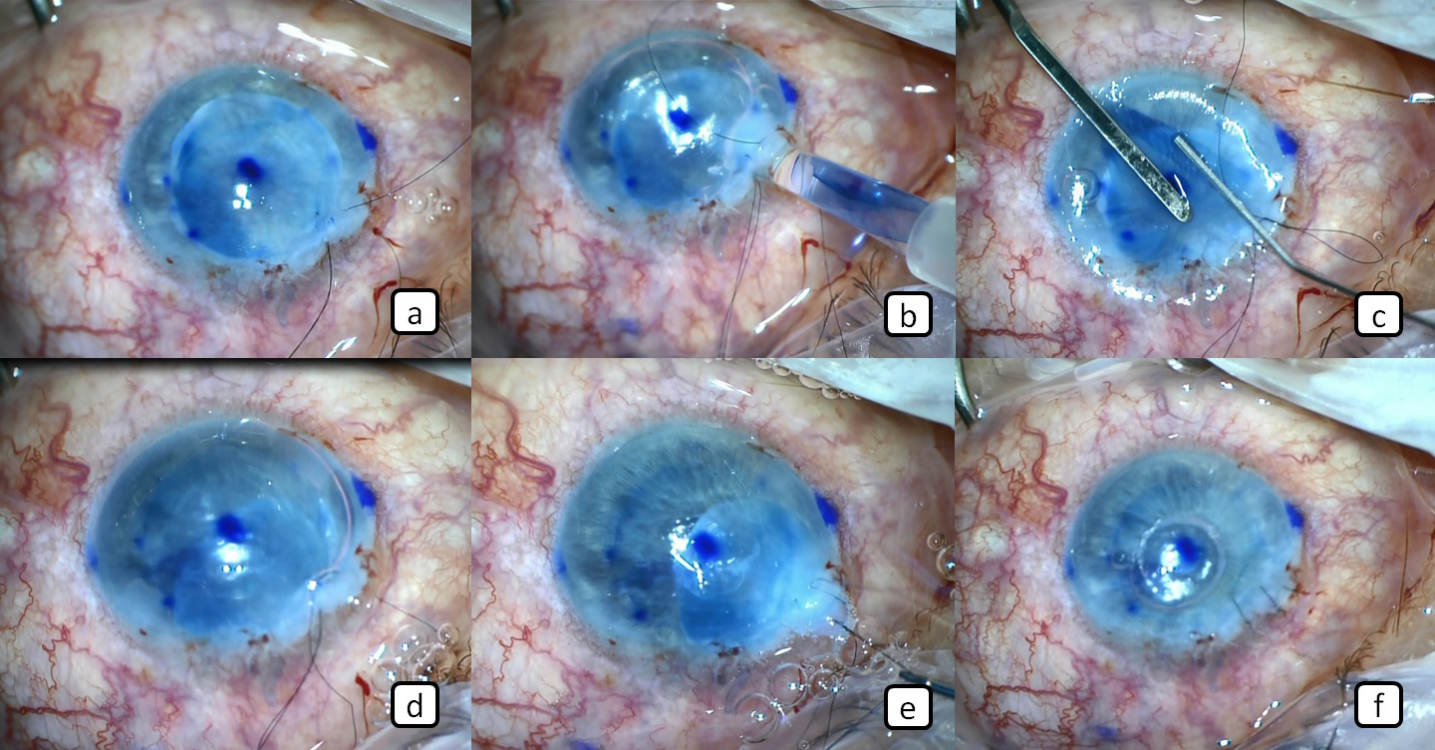
a) The posterior donor stromal tissue barrier was implanted into the anterior chamber. b) The endothelial graft was injected into the anterior chamber. c) The endothelial graft was deployed by gently tapping on the corneal surface. d) The air tamponade was injected into the anterior chamber. e) Posterior donor stromal tissue barrier was removed from the anterior chamber. f) Final appearance of the endothelial graft in the eye at the end of the surgery.

A 3 mm clear corneal incision and 2 side incisions were performed. Intraoperative miosis was achieved by injecting %0.01 carbachol solution (Miostat, Alcon Laboratories Inc., San Diego, USA) into the anterior chamber. The center of the recipient cornea was marked with a 8 mm marker, which was stained by a marker pen to define the size of Descemetorhexis area. A cohesive ophthalmic viscosurgical device (OVD; Healon GV; Abbott Medical Optics, Abbott Park, Illinois, USA) was injected into the anterior chamber to form space and the Descemet’s membrane of the recipient was peeled and removed from the central 8 mm of the recipient cornea (Figure 1c (Fig. 1)). The prepared donor stromal tissue was placed into the anterior chamber through the main incision by the help of a vitreoretinal forceps (Figure 1f (Fig. 1), Figure 2a (Fig. 2)). The OVD was later removed by bimanual irrigation and aspiration and the endothelial graft was injected into the anterior chamber (Figure 2b (Fig. 2)). The pressure in the anterior was lowered during this procedure by allowing the leakage of aqueous humour through the side incisions to cause shallowing of the anterior chamber and ocular hypotony during the insertion process. The injector was gently removed while the wound center was externally compressed with the tip of a 27G anterior chamber cannula to prevent the escape of the endotelial graft from the same incision with the injector. The graft was deployed by gently tapping on the corneal surface (Figure 2c (Fig. 2)). After proper opening and placement of the endothelial graft, verification air was injected into the anterior chamber to ensure proper placement of the graft (Figure 2d (Fig. 2)). After 10 minutes waiting for the endothelial graft to attach on the recipient stroma, the anterior chamber was filled again with balanced salt solution (BSS, Miray Medikal, Bursa, Turkey). The donor posterior stromal tissue was then removed from the anterior chamber through the main incision with the help of a vitreoretinal forceps (Figure 2e (Fig. 2)). The main incision was sutured with a 10-0 nylon suture (Figure 2f (Fig. 2)). Finally, the anterior chamber was filled again with air tamponade and the surgery was completed without any complication. We also did not observe any postoperative complications during the routine postoperative 1-month follow-up.

## Discussion

DMEK is a relatively new technique of posterior lamellar keratoplasty that allows the anatomic replacement of the dysfunctional recipient endothelium without changing the stroma of the recipient cornea. DMEK surgery has many advantages including faster recovery, better postoperative vision, and reduced immune rejection rate, but it is a technically demanding surgery both during graft tissue preparation and transplantation (especially during the unfolding phase) [1], [2]. DMEK surgery in aphakic and vitrectomized eyes is even more difficult due to the absence of support of the iris-lens diaphragm and vitreous humour [3]. It offers several challenges requiring modification of the surgical technique. Although there are a number of studies about DSAEK (Descemet’s stripping automated endothelial keratoplasty) surgery in aphakic and vitrectomized eyes [4], [5], [6], [7], there was only one report of DMEK in the same patient group [3]. 

Afshari et al. [4] reported that eight posterior graft dislocations occurred in their DSAEK surgery. All of their patients had a history of vitrectomy. Five of them had suture-fixated posterior chamber intraocular lenses (IOL), 1 eye had a sulcus-fixated IOL, and two eyes were aphakic. Titiyal et al. used the pars plana infusion during DSAEK surgery in an aphakic and vitrectomized eye to overcome the above-mentioned difficulties [5]. They stated that pars plana infusion was successful in preventing a collapse of the eye during surgical manipulations and also restricted the posterior dislocation of the donor endothelium and air tamponade material.

Groat et al. [7] performed occlusive pupilloplasty and sutured DSAEK graft in order to ensure that the graft would not fall into the vitreous cavity. This technique is not applicable in DMEK, as the endothelial graft tissue in DMEK is significantly thinner compared to DSAEK and even a single suture can easily damage it. 

There is only one report on DMEK in an aphakic and vitrectomized eye [3]. Ozmen and Ozdemir reported that they could successfully perform DMEK surgery with pars plana infusion in an aphakic and vitrectomized patient with the loss of iris tissue in the superior quadrant [3]. The authors could successfully insert and unfold the DMEK graft under pars plana infusion and the graft remained successfully attached at the postoperative 3^rd^ month examination [3].

In the current case, we performed a new surgical technique for DMEK surgery in an aphakic and vitrectomized eye. Here, we used the posterior stroma of the donor cornea as a scaffold and barrier to stabilize the anterior chamber during the surgery. This approach allowed a stable anterior chamber during the surgery and kept the air bubble in the anterior chamber to allow the graft tissue to adhere better on the recipient stroma. Even in the absence of a lens and vitreous support, the injected donor stromal tissue could provide a good physical barrier to prevent complications and contribute to the success of surgery. The placement of a safety suture on this temporary barrier tissue during the surgery provided an extra level of security by preventing the risk of posterior displacement. Therefore, we believe that this technique is a safer approach during DMEK surgery in aphakic and vitrectomized eyes compared to the previously defined pars plana infusion method.

In conclusion, the temporary placement of a half-thickness posterior donor stromal tissue over the iris and pupilla allowed a safer DMEK surgery and very good anterior chamber stability in an aphakic and vitrectomized eye.

## Notes

### Competing interests

The authors declare that they have no competing interests.
